# Role of Oxidative Stress and Inflammation in Postoperative Complications and Quality of Life After Laryngeal Cancer Surgery

**DOI:** 10.3390/cells13231951

**Published:** 2024-11-23

**Authors:** Andjela Zivkovic, Ana Jotic, Ivan Dozic, Simona Randjelovic, Ivana Cirkovic, Branislava Medic, Jovica Milovanovic, Aleksandar Trivić, Aleksa Korugic, Ivan Vukasinović, Katarina Savic Vujovic

**Affiliations:** 1 Faculty of Medicine, University of Belgrade, Dr Subotica 1, P.O. Box 38, 11129 Belgrade, Serbia; zivkovi.angela340@gmail.com (A.Z.); ana.jotic@med.bg.ac.rs (A.J.); ivana.cirkovic@med.bg.ac.rs (I.C.); jmtmilov@gmail.com (J.M.); drcole71.at@gmail.com (A.T.); 2 Clinic for Otorhinolaryngology and Maxillofacial Surgery, University Clinical Center, Serbia Pasterova 2, 11129 Belgrade, Serbia; simonarandjelovic@gmail.com (S.R.); korugicaleksa@gmail.com (A.K.); 3 General and Oral Biochemistry, School of Dental Medicine, University of Belgrade, Dr Subotica-Starijeg 1, 11000 Belgrade, Serbia; ivan.dozic@stomf.bg.ac.rs; 4 Institute of Microbiology and Immunology, Faculty of Medicine, University of Belgrade, Dr Subotića 1, 11000 Belgrade, Serbia; 5 Department of Pharmacology, Clinical Pharmacology and Toxicology, Faculty of Medicine, University of Belgrade, Dr Subotica 1, P.O. Box 38, 11129 Belgrade, Serbia; brankicamedic@gmail.com; 6 Department of Neuroradiology, University Clinical Center, Serbia, Pasterova 2, 11129 Belgrade, Serbia; vukasinovic_i@yahoo.co.uk

**Keywords:** laryngeal cancer, oxidative stress, quality of life, postoperative complications

## Abstract

(1) Background: Laryngeal surgery due to carcinoma leads to significant tissue disruption, cellular injury, and inflammation. This leads to increased levels of reactive oxygen species (ROS), causing oxidative damage that can influence quality of life (QOL) and recovery and complicate the postoperative course. The aim of this study was to compare how postoperative quality of life and surgical complication occurrence interacted with the biomarker levels of oxidative stress (malondialdehyde, MDA; superoxide dismutase, SOD; glutathione peroxidase 1, GPX1; and catalase, CAT) and inflammation (interleukin 1, IL-1; interleukin 6, IL-6; C-reactive protein, CRP) in patients treated with conservative and radical laryngeal surgery. (2) Methods: The study included 56 patients who underwent surgical treatment for laryngeal cancer. Blood samples were collected to analyze oxidative stress and inflammation parameters before surgery and on the first and seventh days postoperatively. Serum concentrations of MDA, SOD, GPX, CAT, IL-1, IL-6, and CRP were measured using coated enzyme-linked immunosorbent assay (ELISA) kits. EORTC QLQ-H&H43 questionnaire was used to measure the QOL of patients. (3) Results and Conclusions: T stage, pain intensity, and the extent of the surgical procedure were established as significant predictive factors for QOL in multivariate analysis. There was a significant positive correlation between surgical complication occurrence and preoperative values of GPX and MDA, but significant predictors of surgical complication occurrence on the 7th postoperative day were SOD and MDA values (*p* < 0.05).

## 1. Introduction

Laryngeal cancer is considered the most common head and neck malignancy, and is ranked 20th in incidence and 18th in mortality compared to all other diagnosed malignancies [1]. The incidence and prevalence of the disease have been rising globally during the past three decades; however, in urban regions with higher socioeconomic status and changes in smoking and alcohol intake, the incidence decreased [2,3].

Since successful oncological treatment requires eradication of the disease, great efforts were made to preserve the larynx and its function during treatment protocols. Surgery remains the first treatment choice in some world regions, compared to radio- or chemotherapy. Surgical treatment of laryngeal carcinoma ranges from more conservative procedures such as cordectomy and partial laryngectomy to more extensive, radical surgeries such as total laryngectomy or pharyngolaryngectomy with unilateral or bilateral neck dissection [4].

The evolution of oxidative stress and inflammation during the postoperative course after laryngeal cancer surgery is an exciting and novel area of investigation. Surgical trauma, especially in extensive procedures, involves significant tissue disruption, cellular injury, and inflammation leading to activation of the complement system, polymorphonuclear cells, and macrophages. These cells release increased levels of reactive oxygen species (ROS), which result in oxidative damage during the postoperative period [5]. Oxidative stress can significantly impact the healing process and pain management after surgery. Excessive ROS can cause further cellular damage and prolonged inflammation, which delays wound healing and can lead to postoperative complications [6,7,8,9]. The extent of the surgery often significantly influences the quality of life (QOL) and can add to postoperative stress [10]. We hypothesized that the more extensive surgery and surgical complications cause more severe oxidative stress and inflammation in surgically treated patients. 

The aim of this study was to compare how postoperative QOL and surgical complication occurrence interact with the levels of biomarkers of oxidative stress (malondialdehyde, MDA; superoxide dismutase, SOD; glutathione peroxidase 1, GPX1; and catalase, CAT) and inflammation (interleukin 1, IL-1; interleukin 6, IL-6; C-reactive protein, CRP) in patients treated with conservative and radical laryngeal surgery. 

## 2. Materials and Methods

### 2.1. Patient Selection

A prospective study included 56 patients with surgically treated laryngeal squamocellullar carcinoma from October 2022 to May 2024 at the tertiary referral center. This study was approved by the Institutional Ethics Committee (745/5-22), and all patients signed the informed consent form prior to their inclusion in the study. The study was registered at ClinicalTrials.gov (ID NCT05857202). The diagnosis of laryngeal carcinoma was established through an otorhinolaryngology clinical examination and laryngomicroscopic examination, followed by a biopsy and histopathological tissue analysis. Additional diagnostic tests included chest radiography, neck computed tomography (CT), and abdominal ultrasonography. The study included patients with surgically treated T1–T4 and N0–N3 laryngeal carcinoma that were not previously treated for malignancies. Patients were excluded if they had inoperable malignant disease, distant metastases, a history of previous malignancies, severe neurological or other comorbidities preventing surgical treatment, substance abuse issues, or were unable to provide informed consent. The treatment approach for each patient was determined by the Oncological Board. Surgical treatment involved tumor resection, with or without neck dissection. Patient data, including demographic, clinical, and histopathological characteristics (age, sex, tobacco use, tumor grade, TNM classification, and treatment modality), were documented. Surgical treatment was classified as reconstructive if cordectomy or partial laryngectomy was performed, and as radical if total laryngectomy, pharyngolaryngectomy with or without defect reconstruction, and/or neck dissections were conducted. A cordectomy involved removing part or all of the vocal cord, and partial laryngectomy involved the removal of one or two laryngeal subregions (supraglottis, glottis or subglottis) while preserving laryngeal function. The extent of tissue removal varies depending on the tumor’s location and size. Total laryngectomy involved removal of the entire larynx and the surrounding structures, while pharyngolaryngectomy involved removal of the entire larynx and a part or complete hypopharynx, depending on the disease extent. It resulted in permanent tracheostomy, due to separation of the airway from the digestive tract. Neck dissection involved removal of neck lymph nodes and surrounding tissue. The extent of the dissection varied, ranging from selective removal of specific lymph node groups to more extensive radical procedures including the removal of muscles, nerves, and blood vessels, depending on the stage of regional metastatic disease.

### 2.2. Pain Assessment

Intensity of pain was measured by the visual analog scale (VAS) [11], where participants self-reported their pain levels by marking a point on a 10 cm line. This scale ranges from 0 cm, representing “no pain”, to 10 cm, representing “the worst possible pain”. Pain levels were assessed before surgery and at two intervals after surgery: on the 1st and 7th postoperative day. VAS scores were classified as follows: 0 to 4 mm indicating no pain, 5 to 44 mm indicating mild pain, 45 to 74 mm indicating moderate pain, and 75 to 100 mm indicating severe pain [12].

### 2.3. Quality of Life

The Serbian version of the head and neck-specific quality of life questionnaires of the European Organization for the Research and Treatment of Cancer (EORTC QLQ-H&H43) was used to measure the quality of life of patients in the study preoperatively and on the 7th postoperative day [13,14]. The questionnaire includes 12 multi-item scales (dry mouth/sticky saliva, pain in the mouth, senses, social eating, swallowing, sexuality, body image, speech problems, problems with teeth, anxiety, shoulder problems, and skin problems) and 7 single-item symptom scales (coughing, opening mouth, social contact, neurological problems, swelling of the neck, weight loss, and problems with wound healing). All items have a Likert-like response format (not at all  =  1; a little  =  2; quite a bit  =  3; and very much  =  4). Eleven scale and single-item scores are calculated by converting raw scores into a 0–100 scale, where a score of 100 indicates a high symptom burden. Since the questionnaire is self-reported, patients completed it either on their own or were guided by medical staff due to age-related difficulties or functional illiteracy.

### 2.4. Measurement of the Oxidative Stress and Inflammatory Parameters

Oxidative stress and inflammation parameter analysis was performed on blood samples collected from patients before surgery, and on the 1st and 7th postoperative day. The serum levels of inflammatory markers, including IL-1 (pg/mL), IL-6 (pg/mL), and CRP (ng/mL), as well as oxidative stress markers, such as GPX (pg/mL), SOD (pg/mL), MDA (ng/mL), and CAT (U/mL), were measured using coated enzyme-linked immunosorbent assay (ELISA) kits following the manufacturer’s instructions (Elabscience, Wuhan, China). The ELISA kits for IL-1 and IL-6 were based on the Sandwich ELISA method, with plates pre-coated with antibodies specific to human cytokines. Optical density (OD) readings were taken at 450 nm using a Multiskan EX plate reader (Thermo Fisher Scientific, Vantaa, Finland). Sample concentrations were calculated by comparing the OD of the samples to a standard curve generated with GraphPad Prism 9.0 software (GraphPad Software Inc., San Diego, CA, USA).

### 2.5. Statistical Analysis

Numerical data were presented as mean with 95% confidence interval. Categorical variables were summarized by absolute numbers with percentages. The normality of data was assessed using graphical (histogram, box plot) and mathematical (Shapiro–Wilk, skewness and kurtosis, and coefficient of variation) methods. According to the chosen error of the first type of 0.05, effect size of 0.35, and total sample size of 56, with two groups, three measurements, and correlation among representative measures of 0.7, the post hoc power of the study is 82%. The calculation was performed using G Power ver.3.1.9.4. Correlations between oxidative stress, inflammation parameters, complication occurrence, and QOL measured preoperatively and on the 1st and 7th day postoperatively were assessed by Pearson correlation coefficient. Univariate and multivariate linear regression analysis was performed to identify predictors of complication occurrence and QOL measured preoperatively and on the 1st and 7th day postoperatively. In all analyses, the significance level was at 0.05. Statistical analysis was performed using IBM SPSS statistical software (SPSS for Windows, release 25.0, SPSS, Chicago, IL, USA).

## 3. Results

### 3.1. Characteristics of Study Population and Carcinoma-Related Characteristics

A total of 56 patients with laryngeal carcinoma were included in the study. Patients were predominantly male (87.5%), with a mean age of 65.2 ± 7.5 years. The majority of patients were smokers (87.5%) and had one comorbidity (42.9%). Among the study population, 69.6% had cancer histologically classified as grade II, more than half (51.8%) had radical surgical procedure, 64.3% required tracheotomy, and 18 (32.1%) experienced postsurgical complications. Five (8.92%) patients had pharyngocutaneus fistula, five (8.92%) had bleeding that required revision surgery, four (7.12%) patients had myocardial ischemia, three (5.35%) had pulmonary embolism, and one (1.78%) had pneumomediastinum. Characteristics of the study population and carcinoma-related characteristics are shown in Table 1.

### 3.2. Parameters of Oxidative Stress and Inflammation During Preoperative and Postoperative Period

**Figure 1:**
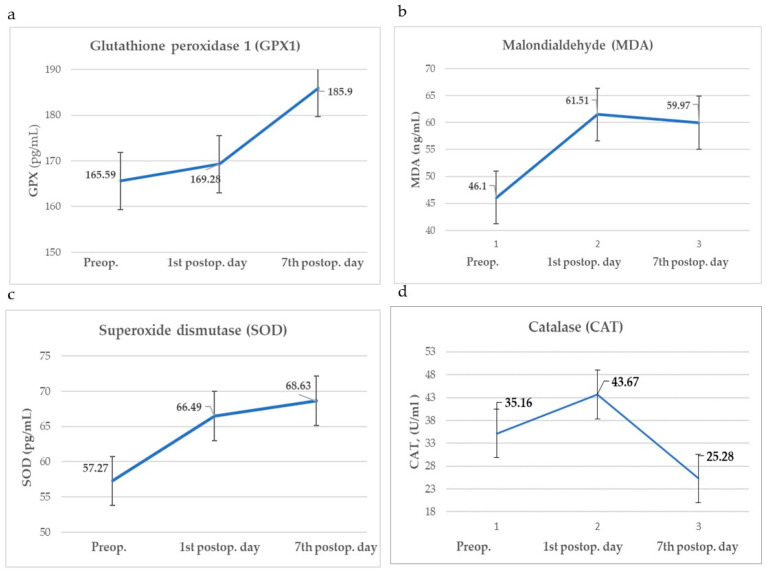
Parameters of oxidative stress: (**a**) GPX1, (**b**) SOD, (**c**) MDA, (**d**) CAT.

**Figure 2:**
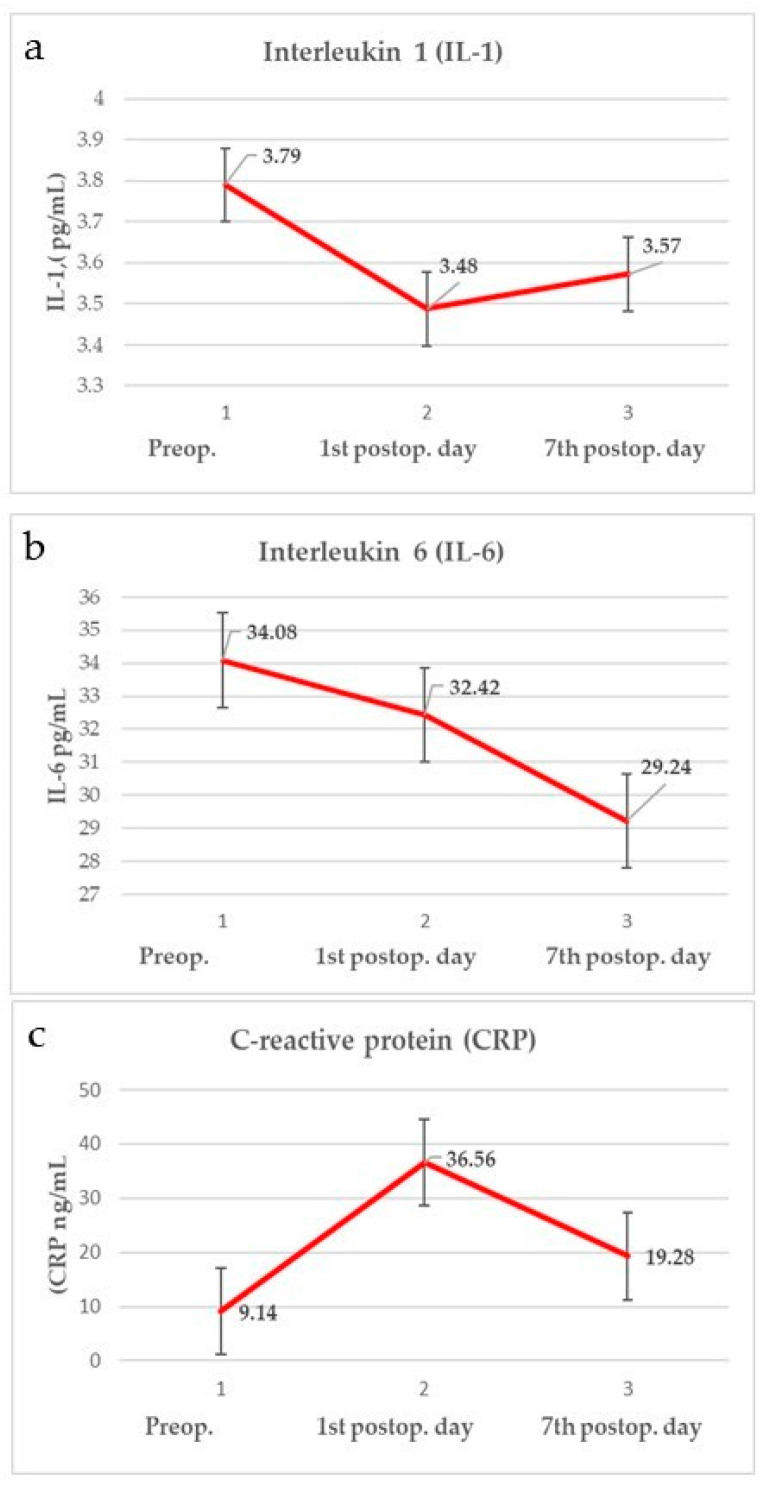
Parameters of inflammation: (**a**) IL-1, (**b**) IL-6, (**c**) CRP.

**Figure 3:**
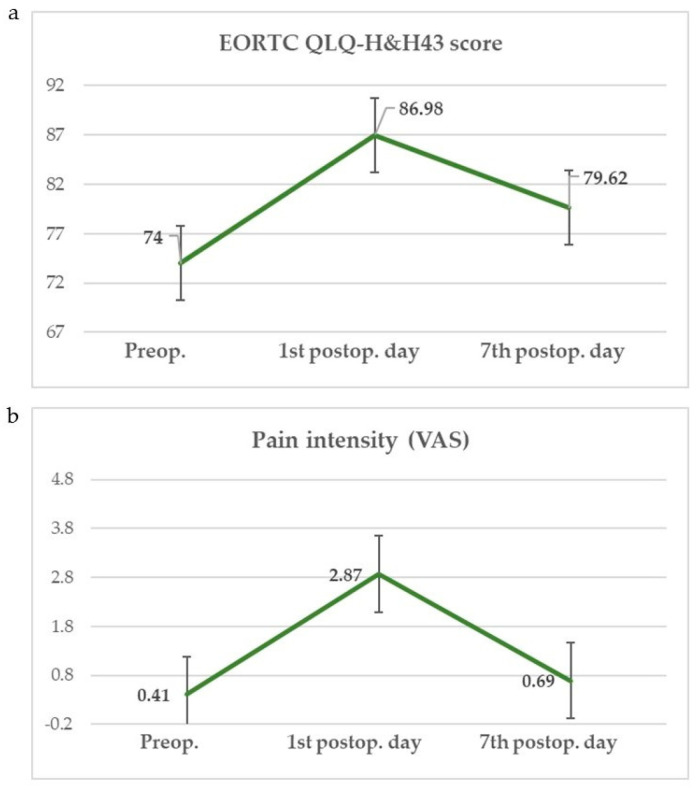
EORTC QLQ—H&N43 score (**a**) and pain intensity (VAS) (**b**).

Parameters of oxidative stress and inflammation measured during the preoperative and postoperative period (1st and 7th postoperative day) are shown in Figure 1 and Figure 2. Values of the EORTC QLQ—H&N43 questionnaire and pain intensity (VAS) measured preoperatively and on the 1st and 7th postoperative day are shown in Figure 3.

### 3.3. Correlation Between Demographic and Clinical Characteristics; Oxidative Stress, Inflammation Biomarkers and the EORTC QLQ—H&N43 Score

Numerous clinical characteristics were significantly correlated with the EORTC QLQ—H&N43 score (Table 2). There was a weak positive correlation between the EORTC QLQ—H&N43 score preoperatively and stage (r(56) = 0.108, *p* < 0.001), extent of the surgical procedure (r(56) = 0.335, *p* = 0.012), pain intensity (r(56) = 0.377, *p* = 0.004), and IL-1 levels (r(56) = 0.326, *p* = 0.014). On the 1st postoperative day, there were weak to moderate correlations between the number of comorbidities (r(56) = 0.290, *p* = 0.030), T stage (r(56) = 0.345, *p* = 0.009), the extent of the surgical resection (r(56) = 0.365, *p* = 0.006), previously performed tracheostomy (r(56) = 0.295, *p* = 0.027), pain intensity (r(56) = 0.495, *p* < 0.001), and EORTC QLQ—H&N43 score. On the 7th postoperative day, only the extent of surgical resection was strongly positively correlated with heavier symptom burden in patients according to the EORTC QLQ—H&N43 score (r(56) = 0.605, *p* < 0.001).

### 3.4. Univariate and Multivariate Linear Regression Analysis with the EORTC QLQ—H&N43 Score as a Dependent Variable

**Figure 4:**
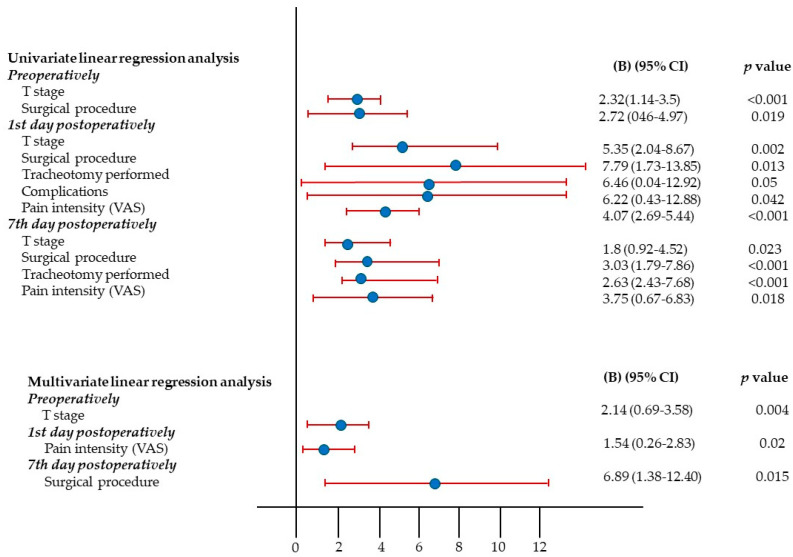
Univariate and multivariate linear regression analysis with the EORTC QLQ-H&N43 score as a dependent variable.

Univariate and multivariate linear regression analysis with the EORTC QLQ—H&N43 score as a dependent variable is shown in Figure 4. In univariate linear regression analysis, preoperatively significant predictors of the EORTC QLQ—H&N43 score were T stage (*p* < 0.001) and the extent of the surgical procedure (*p* = 0.019). On the 1st postoperative day, significant predictors of the EORTC QLQ—H&N43 score were T stage, the extent of the surgical procedure, tracheotomy performed before surgery, postoperative complication occurrence, and pain intensity (*p* < 0.05). On the 7th postoperative day, significant predictors of the EORTC QLQ—H&N43 score were T stage, the extent of the surgical procedure, tracheotomy performed before surgery, and pain intensity (*p* < 0.05).

Multivariate regression analysis used the preoperative EORTC QLQ–H&N43 score as the dependent variable. Preoperatively, the overall model was significant, F(2, 53) = 7.753, *p* = 0.001, accounting for 22.6% of the variance in EORTC QLQ–H&N43 scores (R^2^ = 0.226, Adjusted R^2^ = 0.197). The T stage was a significant predictor (B = 2.14, 95% CI [0.696, 3.584], *p* = 0.004), indicating that patients with a higher T stage scored higher on the EORTC QLQ–H&N43 questionnaire.

On the first postoperative day, a separate multivariate regression analysis showed that the overall model was significant, F(5, 50) = 5.226, *p* = 0.001, explaining 34.3% of the variance in EORTC QLQ–H&N43 scores (R^2^ = 0.343, Adjusted R^2^ = 0.278). Pain intensity, measured by the visual analog scale (VAS), was a significant predictor (B = 1.545, 95% CI [0.258, 2.833], *p* = 0.02), suggesting that patients with higher pain intensity also scored higher on the EORTC QLQ–H&N43.

On the 7th postoperative day, multivariate regression analysis showed a significant overall model, F(6, 49) = 5.226, *p* < 0.001, which explained 49.1% of the variance in EORTC QLQ–H&N43 scores (R^2^ = 0.491, Adjusted R^2^ = 0.429). The extent of surgical resection was a significant predictor (B = 6.893, 95% CI [1.384, 12.403], *p* = 0.015), indicating that patients who underwent more extensive surgery scored higher on the EORTC QLQ–H&N43 compared to those who had conservative surgery.

### 3.5. Correlation Between Demographic and Clinical Characteristics; Oxidative Stress and Inflammation Biomarkers and Complication Occurrence

There was a weak positive correlation between complication occurrence and age (r(56) = 0.312, *p* = 0.019), and the number of comorbidities in patients included in the study (r(56) = 0.279, *p* = 0.038) (Table 3).

Preoperatively, there was a weak positive correlation between the occurrence of complications and GPX (r(56) = 0.343, *p* = 0.01) and MDA levels (r(56) = 0.363, *p* = 0.006) (Table 4). On the 1st postoperative day, SOD levels showed a weak positive correlation (r(56) = 0.375, *p* = 0.004), and MDA levels had a moderate positive correlation (r(56) = 0.484, *p* < 0.001) with complications. By the 7th postoperative day, GPX (r(56) = 0.315, *p* = 0.018) and SOD levels (r(56) = 0.394, *p* = 0.003) showed weak positive correlations, but MDA levels had a strong positive correlation (r(56) = 0.798, *p* < 0.001) with complications.

### 3.6. Univariate and Multivariate Linear Regression Analysis with Complication Occurrence as Dependent Variable

**Figure 5:**
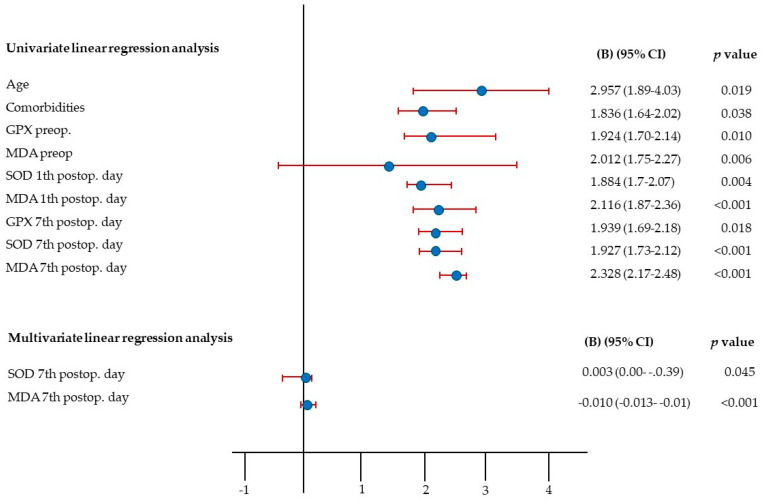
Univariate and multivariate linear regression analysis with complication occurrence as a dependent variable.

Figure 5 presents the univariate and multivariate linear regression analysis with complication occurrence as the dependent variable. In the univariate analysis, significant predictors of postoperative complications included the age, the presence of comorbidities, and preoperative levels of GPX and MDA, SOD and MDA levels on the 1st postoperative day, and GPX, SOD, and MDA levels on the 7th postoperative day (all *p* < 0.05).

In the multivariate regression analysis, the overall model was significant, F(9, 46) = 11.127, *p* < 0.001, explaining 68.5% of the variance in complication occurrence (R^2^ = 0.685, Adjusted R^2^ = 0.624). SOD levels on the 7th postoperative day were a significant predictor (B = 0.003, 95% CI [−0.00, −0.065], *p* = 0.045), indicating that elevated SOD levels were associated with postoperative complications. Similarly, MDA levels on the 7th postoperative day were also significant (B = −0.1, 95% CI [−0.013, −0.007], *p* < 0.001), suggesting that higher MDA levels were linked to complications.

**Figure 6:**
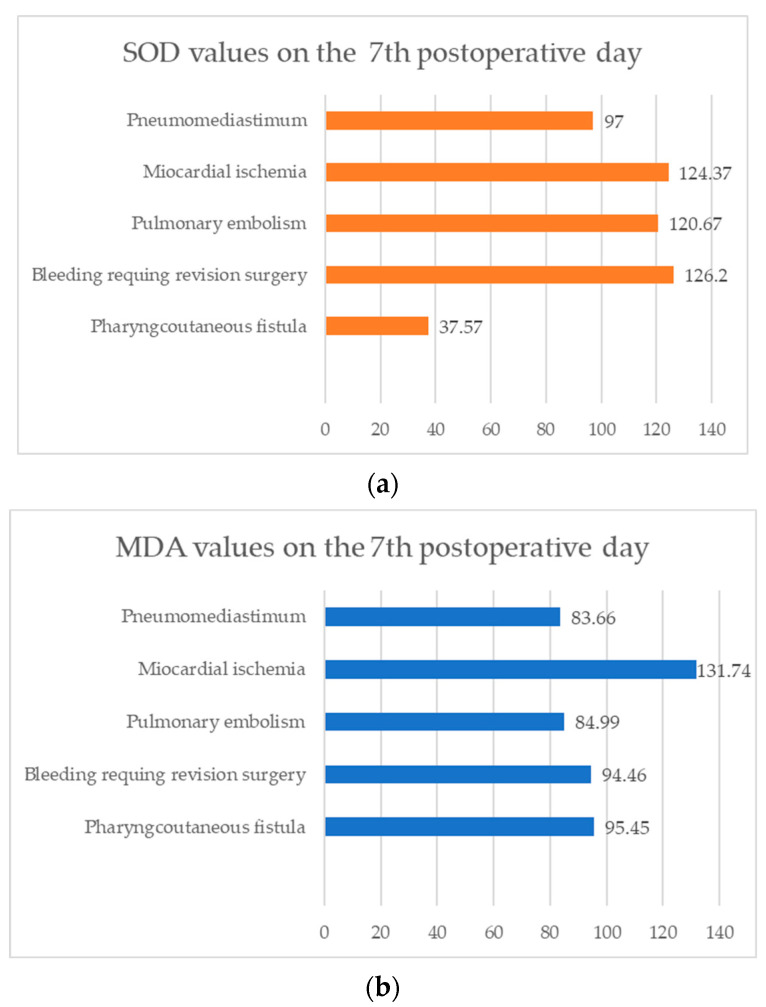
SOD values (**a**) and MDA values (**b**) on the 7th postoperative day depending on postoperative complications.

Figure 6a,b show that the SOD and MDA levels on the 7th postoperative day depended on the type of complication. Both parameters were elevated, with variations across different complications. During myocardial ischemia, the levels of both parameters were especially high, followed by other life-threatening complications, such as pulmonary embolism, pneumomediastinum, bleeding requiring revision surgery, and pharyngocutaneous fistula formation. Interestingly, while pharyngocutaneous fistula is considered a challenging surgical complication, it was not accompanied by particularly high levels of either parameter.

## 4. Discussion

This research is the first to explore and establish a correlation between postoperative complications, QOL, and oxidative stress and inflammation biomarkers in patients surgically treated for laryngeal cancer. Our previous research focused on pain modulation postoperatively and on its influence on oxidative stress and inflammation parameters in patients with surgically treated laryngeal cancer [15]. This research examined whether postoperative complications could be predicted based on the levels of these biomarkers.

In recent years, there has been an increasing focus on minimizing the negative impacts of surgery and preventing the onset of secondary conditions to enhance patients’ QOL. These efforts aim to alleviate the financial strain on healthcare systems, which often arises from extended hospital stays and additional corrective treatments. Surgical procedures can impose varying physiological stress levels on the body, potentially resulting in postoperative complications [9]. Surgery is a critical event that triggers considerable inflammation and ischemia/reperfusion injury (IRI), leading to increased production of ROS and a decline in antioxidant defense mechanisms, ultimately causing oxidative stress [16]. MDA is a pro-oxidant and frequently used biomarker for assessing oxidative stress and lipid peroxidation [17]. Conversely, antioxidant enzymes such as SOD, GPX1, and CAT play a crucial role in neutralizing free radicals, serving as the primary defense against tissue damage induced by ROS [18].

Pro-inflammatory cytokines (IL-1 and IL-6) are released in response to tissue injury, inflammation, and infection. Regarding inflammation status, surgery can trigger systemic immunological and inflammatory responses. This includes the activation of various immune cells such as polymorphonuclear leukocytes, macrophages, monocytes, platelets, and mast cells. Additionally, surgery can lead to the excessive production of cytokines, which further contributes to the inflammatory response [19]. During surgical procedures, the immune system activates and releases these cytokines, which can increase the production of ROS and intensify oxidative stress. IL-1 and IL-6 are involved in the inflammatory response following tissue damage and contribute to hyperalgesia [20]. Additionally, CRP is an acute-phase protein of the innate immune system that generates pro-inflammatory cytokines and amplifies the inflammatory response [21].

When managing head and neck cancers (HNCs), QOL is a crucial factor to consider as it shapes treatment decision-making and guides postoperative rehabilitation to achieve the general well-being of a patient. Advanced tumors, extensive surgical resections, the need for flap reconstruction, neck dissections, and postoperative radiation therapy are all linked to poorer QOL outcomes [22]. T stage, pain intensity, and the extent of the surgical procedure were established as significant predictive factors of QOL in multivariate analysis.

Our study failed to establish a significant correlation between QOL, oxidative stress, and inflammatory biomarkers. Only a handful of studies have examined the connection between oxidative stress and QOL. Increased lipid peroxidation and reductions in antioxidant levels in more advanced diseases were correlated with poorer QOL in patients with diabetes mellitus and fibromyalgia [23,24]. Research on a larger number of patients with laryngeal cancer could provide more data on the matter. Future research should prioritize the integration of QOL assessments in randomized trials, ensure results are reported according to established guidelines, and apply knowledge translation principles to optimize the use of QOL data by both clinicians and patients [25].

Complications occurred in 31.2% of the patients. As expected, a positive correlation was established between postoperative complications and age and the number of comorbidities in patients with laryngeal cancer. The most common was the formation of pharyngocutaneus fistula, followed by myocardial ischemia, postoperative bleeding, and pneumothorax, which was similar to the literature data [26,27]. Inflammatory parameters examined in the study did not correlate with the occurrence of postoperative complications, though some previous studies have reported the opposite [6,28,29,30,31]. A significant predictor of surgical complication occurrence on the 7th postoperative day was elevated values of SOD and MDA. Elevated values of oxidative stress parameters postoperatively indicate that antioxidant reserve was decreased which caused dysfunction in the ROS elimination system, development of inflammation, and further increased production of excessive ROS. This may contribute to postoperative complication occurrence and delayed recovery, which was already confirmed in patients who underwent esophageal, liver, and prostate surgery [32,33]. Elevated oxidative stress markers were also detected in previous research in life-threatening events such as myocardial ischemia and pulmonary embolism. The oxidative stress response mechanism is explained by ischemia–reperfusion (IR) tissue injury. During ischemia, a shortage of oxygen and nutrients impairs mitochondrial function in myocardial and vascular endothelial cells [34,35]. During reperfusion, a surge of ROS is generated, further damaging mitochondrial structures and functions, and triggering an inflammatory response. SOD and GPX initially increase in response to mitochondrial damage as part of the antioxidant defense system; however, their levels and activity may decrease if oxidative stress persists in case of complication occurrence. MDA levels increase due to excess ROS production, signaling greater oxidative damage to cells and mitochondrial membranes [36]. Our study is the only one in the current literature examining the influence of oxidative stress and inflammation on postoperative complication occurrence in patients with laryngeal cancer. Our unique findings can only be analyzed and compared with available results from other groups of cancer patients or those experiencing similar complications.

Another important finding was a significant positive correlation between surgical complication occurrence and preoperative GPX and MDA values. Patients with higher preoperative oxidative stress markers can experience even greater oxidative stress triggered by surgery, making them prone to prolonged inflammation, delayed postoperative recovery, and severe complications [37]. This could indicate that the measurement of preoperative oxidative parameters can be potentially used in predicting the postoperative course in patients after laryngeal surgery.

This study has several strengths. It is the first to investigate the relationship between postoperative QOL and the occurrence of surgical complications, with oxidative stress biomarker levels in patients following laryngeal cancer surgery. This is also the only study that has shown that preoperative levels of oxidative stress enzymes can indicate potential postoperative complications. A limitation of the study is the relatively small sample size, especially subgroups undergoing conservative and radical surgery. A larger patient cohort would further validate our findings.

## 5. Conclusions

Clinical characteristics which include T stage, pain intensity, and extent of surgical procedure were established as significant predictive factors of QOL. None of the oxidative stress and inflammation parameters predicted reported QOL scores. Preoperative values of GPX and MDA were significantly positive and correlated with surgical complication occurrence, while significant predictors of surgical complication occurrence postoperatively were elevated values of SOD and MDA.

While the link between oxidative stress, inflammation, and events during the postoperative course in laryngeal cancer patients is emerging, more research is needed to fully understand how best to address oxidative stress in clinical practice. Future studies exploring the efficacy of antioxidants in reducing postoperative complications and improving QOL, as well as understanding the mechanisms behind ROS-induced events, are crucial for developing optimized management strategies.

## Figures and Tables

**Table 1:** Study population and carcinoma-related characteristics.

Variable	n (%)
Sex	
Male	49 (87.5)
Female	7 (12.5)
Age, mean ± SD	65.2 ± 7.5
Smoking	
No	7 (12.5)
Yes	49 (87.5)
Comorbidities	
No	17 (30.4)
One	24 (42.9)
Two	13 (23.2)
Three and more	2 (3.6)
Carcinoma localization	
1 subregion	15 (26.8)
2 subregions	12 (21.4)
2 or 3 subregions with extralaryngeal expansion	29 (51.8)
Histological grade	
0	7 (12.5)
1	6 (10.7)
2	39 (69.6)
3	4 (7.1)
TNM staging	
T stage	
1	20 (35.7)
2	21 (37.5)
3	12 (21.4)
4	3 (5.4)
N stage	
0	37 (66.1)
1	14 (25.0)
2	2 (3.6)
3	3 (5.4)
M stage	
0	54 (96.4)
1	2 (3.6)
Surgical procedure	
Conservative	27 (48.2)
Radical	29 (51.8)
Tracheotomy performed	36 (64.3)
Postsurgical complications	18 (32.1)

**Table 2:** Significant correlation between demographic and clinical characteristics; oxidative stress and inflammation biomarkers and the EORTC QLQ—H&N43 score.

Variable	EORTC QLQ—H&N43 Score					
	Preoperatively		1st Day Postoperatively		7th Day Postoperatively	
	r	*p*	r	*p*	r	*p*
Comorbidities			0.290	0.030		
T stage	0.108	<0.001	0.345	0.009	0.332	0.012
Surgical procedure	0.335	0.012	0.365	0.006	0.605	<0.001
Tracheotomy Performed			0.295	0.027	0.50	<0.001
Pain intensity (VAS)	0.377	0.004	0.495	<0.001	0.392	0.003
Inflammation						
IL-1	0.326	0.014				
CRP	−0.28	0.037				

**Table 3:** Significant correlation between demographic and clinical characteristics and complication occurrence.

	Complications	
	r	*p*
Age	0.312	0.019
Comorbidities	0.279	0.038

**Table 4:** Significant correlation between pain intensity, oxidative stress and inflammation biomarkers, and complication occurrence.

Variable	Complications					
	Preoperatively		1st Day Postoperatively		7th Day Postoperatively	
	r	*p*	r	*p*	r	*p*
Oxidative stress						
GPX	0.343	0.01			0.315	0.018
SOD			0.375	0.004	0.394	0.003
MDA	0.363	0.006	0.484	<0.001	0.798	<0.001

## Data Availability

Data will be available on request from the corresponding author (katarinasavicvujovic@gmail.com).
